# Association between serum iron concentrations and cognitive impairment in older adults aged 60 years and older: A dose-response analysis of National Health and Nutrition Examination Survey

**DOI:** 10.1371/journal.pone.0255595

**Published:** 2021-08-02

**Authors:** Zonglin Gong, Wenlei Song, Minjun Gu, Xiaoming Zhou, Changwei Tian

**Affiliations:** 1 Department of Integrated Services, Kunshan Centers for Disease Control and Prevention, Kunshan, Jiangsu, China; 2 Department of Disease Control, Kunshan Centers for Disease Control and Prevention, Kunshan, Jiangsu, China; Chinese Academy of Sciences, CHINA

## Abstract

Epidemiological evidence on peripheral iron and cognitive impairment in older adults is sparse and limited. Results on serum iron and cognitive impairment in older adults from the National Health and Nutrition Examination Survey have not been reported. Data on serum iron and cognitive impairment from individuals ≥ 60 years of age were obtained from the 2011–2014 NHANES (N = 3,131). Serum iron concentrations were determined with DcX800 method. Cognitive impairment was assessed with four cognitive tests: the Digit Symbol Substitution Test (DSST), the Animal Fluency (AF), the Consortium to Establish a Registry for Alzheimer’s Disease Delayed Recall (CERAD-DR) and Word Learning (CERAD-WL) tests. Logistic regression and restricted cubic splines were adopted to explore the dose-response relationship between serum iron concentrations and cognitive impairment. Comparing the highest to lowest tertile of serum iron concentrations, the multivariate-adjusted odds ratios of scoring low on the DSST were 0.70 (0.49–1.00), 0.88 (0.65–1.20) for CERAD-WL, 0.65 (0.48–0.88) for CERAD-DR, and 0.78 (0.53–1.15) for AF. Stratified analyses by sex showed that the above-mentioned associations were mainly found in men; however, the interaction with sex was not significant. Dose-response analysis showed that relationships between serum iron and cognitive impairment evaluated by DSST and CERAD-DR were linear, respectively.

## Introduction

It was estimated that 35.6 million people lived with dementia worldwide in 2010, with numbers expected to 65.7 million in 2030 and 115.4 million in 2050 [1]. Dementia affects an estimated 2.4 to 5.5 million individuals in the United States [2], and the number is expected to rise to 13.8 million by 2050 [1]. The prevalence of mild cognitive impairment varies greatly (3%-42%) in older adults worldwide [3]. Iron is essential for normal development and functions of the brain, and plays myriad keystone roles in a number of cellular processes including neurotransmitter synthesis, myelination of neurons, and mitochondrial function [4, 5]. Previous studies showed that brain iron accumulation could have a detrimental effect on cognitive ability because excess of iron could induce oxidative stress, energy failure, synaptic loss and cell death, et al. [6–11]. However, epidemiological evidence on peripheral iron and cognitive impairment incidence is limited [12], and the diversity of the existing evidence precludes any conclusions relating to the relationship between peripheral iron and cognitive impairment incidence [12]. Recently, results from the NHANES showed that total iron intake was inversely associated with cognitive impairment evaluated by Digit Symbol Substitution Test (DSST) [odds ratios (95% confidence intervals): 0.44 (0.21–0.95)], but not with Consortium to Establish a Registry for Alzheimer’s Disease (CERAD) test [1.00 (0.47–2.12)] and Animal fluency (AF) test [0.62 (0.31–1.25)] [13]. However, the association between peripheral iron and cognitive impairment from the NHANES has not been reported. Therefore, the purpose of this study was to examine the relationship between peripheral iron and cognitive impairment.

## Materials and methods

### Data collection

The NHANES is a nationally representative, continuous cross-sectional study of US population. Data from two consecutive NHANES 2-year cycles (2011–2012, 2013–2014) are collected, because these two cycles specifically inquired about cognitive impairment. Individuals who do not provide data of serum iron and cognitive performance tests were excluded. Written informed consent was obtained for all participants or proxies. The survey protocol was approved by the Research Ethics Review Board at the National Center for Health Statistics.

### Cognitive performance tests

Participants aged 60 years and older were eligible for cognitive performance tests, and a series of assessments were introduced in NHANES, including CERAD, AF and DSST. The CERAD is used to assess the ability for new learning, delayed recall and recognition memory, and the test consists of three consecutive learning trials, and a delayed recall. In the CERAD-Word Learning test (CERAD-WL) that consists of 3 consecutive learning trials, participants are instructed to read aloud 10 unrelated words, one at a time, and the order of the 10 words is changed in each of the three learning trials. In the CERAD-Delayed Recall test (CERAD-DR), participant was asked to recall the 10 unrelated words used in the first CERAD-WL trial, after all of the cognitive performance tests were completed (approximately 8–10 minutes from the start of the word learning trials). The AF test examines categorical verbal fluency, a component of executive function. The Digit Symbol Substitution test (DSST), a performance module from the Wechsler Adult Intelligence Scale, relies on processing speed, sustained attention, and working memory.

The assessments were administered by trained interviewers at the beginning of the face-to-face private interview in the Mobile Examination Center. Detailed information on the cognitive performance tests including quality assurance, quality control, data processing and editing are described in the NHANES (https://www.cdc.gov/nchs/nhanes/index.htm). Based on prior literature[14], cutoffs of <14 for AF, <34 for DSST, *<*17 for CERAD-WL and *<*5 for CERAD-DR were used to distinguish potential cognitive impairment from healthy cognitive function and lack of cognitive impairment in the NHANES.

### Serum iron measurement

Serum specimens are processed, stored, and shipped to the Collaborative Laboratory Services, Ottumwa, Iowa for analysis. The DcX800 method used to measure the iron concentration is a timed-endpoint method. In the reaction, iron is released from transferrin by acetic acid and is reduced to the ferrous state by hydroxylamine and thioglycolate. The ferrous ion is immediately complexed with the FerroZine Iron Reagent. The system monitors the change in absorbance at 560 nm at a fixed-time interval. This change in absorbance is directly proportional to the concentration of iron in the sample. The NHANES quality control and quality assurance protocols meet the 1988 Clinical Laboratory Improvement Act mandates.

### Covariates

According to the previously related publications [12, 13], the following covariates were included in this study: data release cycle, age group (60 to <65 y, 65 to <70 y, 70 to <75 y, 75 to <80 y, and ≥80 y), sex, race/ethnicity (Mexican American, Other Hispanic, Non-Hispanic White, Non-Hispanic White, Other Race), body mass index (<25 kg/m^2^, 25 to <30 kg/m^2^, ≥30 kg/m^2^), poverty-income ratio (<1, 1 to 2, >2), education (< 9th grade, 9-11th grade, high school graduate, some college or AA degree, college graduate or above), marital status (never married, married, others), hypertension, diabetes, moderate recreational activities for at least 10 minutes continuously in a typical week, serum cotinine (continuous) and daily intakes (continuous) of sugar, fat, protein, caffeine, alcohol and total energy.

### Statistical analysis

Logistic regression and restricted cubic splines were adopted to explore the dose-response relationship between serum iron concentrations and cognitive impairment. In logistic regression, subjects were classified into tertiles according to their serum iron concentrations, and ORs and 95% CIs of cognitive impairment for subjects in tertile 2 (T2) and tertile 3 (T3) were calculated as compared to those in tertile 1. We calculated three different logistic regression models. Model 1 was adjusted for data release cycle, age group, sex, race/ethnicity and body mass index. Model 2 was adjusted for covariates in model 1, and also poverty-income ratio, education and marital status. Model 3 was adjusted for covariates in model 2, and also hypertension, diabetes, moderate recreational activities, serum cotinine and daily intakes of sugar, fat, protein, caffeine, alcohol and total energy. Tests for trends across categories were performed by modeling serum iron concentration as a continuous variable using the median value of each category. The interaction with sex were tested by using the cross-product term of serum iron and sex. The dose–response relationship between serum iron concentrations and cognitive impairment was assessed using restricted cubic splines with three knots located at the 5th, 50th, and 95th percentiles of serum iron concentrations, and a *P* value for nonlinearity (*P*_for non-linearity_) was calculated by testing the null hypothesis that the coefficient of the second spline is equal to 0 [15]. All analyses were conducted using STATA version 12.0, and *P* ≤ 0.05 was considered statistically significant.

## Results

Table 1 presents characteristics of the study participants. A total of 3,131 older adults aged 60 years and older were included in this study. The mean concentration (SD) of serum iron was 82.80 (31.89) ug/dL. The weighted prevalence of cognitive impairment was 15.32% for DSST, 23.45% for CERAD-WL, 26.16% for CERAD-DR, and 22.83% for AF, respectively. Compared with participants in T1 of serum iron concentrations, those in T3 of serum iron concentrations were more likely to be male (58.58% vs. 37.74%), and have low prevalence of cognitive impairment (DSST: 11.29% vs. 21.17%, DERAD-WL: 20.73 vs. 26.76%, CERAD-DR: 21.81% vs. 30.27%, AF: 19.27% vs. 29.68%), diabetes (20.72% vs. 35.07%), obesity (31.09% vs. 44.62%) and hypertension (73.26% vs. 83.01%).

**Table 1:** Population characteristics by tertiles of serum iron concentrations (mean values: tertile 1: 51.07 ug/dL, tertile 2: 80.10 ug/dL, tertile 3: 119.67 ug/dL).

Characteristics	Overall	Tertile 1	Tertile 2	Tertile 3
Age, year	70.06±6.97	70.38±6.91	69.94±6.93	69.35±6.91
Women, %	51.54	62.26	48.57	41.42
Serum iron (ug/dL)	82.80±31.89	51.07±11.53	80.10±8.16	119.67±25.73
DSST<40, %	15.32	21.17	13.38	11.29
CERAD-WL<17, %	23.45	26.76	22.80	20.73
CERAD-DR<5, %	26.16	30.27	26.51	21.81
AF<14, %	22.83	29.68	20.01	19.27
Diabetes, %	27.28	35.07	27.00	20.72
Obesity, %	37.00	44.62	35.86	31.09
Hypertension, %	77.77	83.01	76.28	73.26
Education, %				
<9^th^ grade	14.95	17.34	13.74	12.49
9-11^th^ grade	14.62	17.24	13.15	12.39
High school graduate	23.02	24.23	22.09	23.05
Some college or AA degree	26.10	23.66	27.74	27.82
College graduate or above	21.09	17.24	23.10	24.26
Marital status, %				
Never married	5.96	5.94	6.33	5.19
Married	53.43	47.80	54.09	60.63
Others	40.61	46.26	39.58	34.18
Race/Hispanic origin (%)				
Mexican American	9.25	8.62	9.27	10.36
Other Hispanic	10.13	10.34	8.94	10.56
Non-Hispanic White	45.37	41.28	48.06	51.17
Non-Hispanic Black	23.98	29.69	24.11	14.42
Other Race	11.26	10.06	9.61	13.50
Ratio of family income to poverty (%)				
<1	19.84	21.99	17.38	16.89
1–2	30.28	32.57	29.85	28.08
2–5	49.88	45.45	52.77	55.03
Physical activity^a^				
Yes	35.88	31.42	39.21	39.49
No	64.10	68.58	60.79	60.51
Daily intake				
Total energy (kcal)	1802.42±691.57	1712.19±673.03	1830.04±698.53	1870.47±681.32
Total sugars (g)	94.79±52.34	91.57±49.52	98.09±53.91	94.38±50.13
Protein (g)	72.20±30.00	68.57±29.00	73.44±30.63	74.91±29.92
Total fat (g)	68.44±33.34	64.89±32.47	70.53±34.36	70.13±32.66
Alcohol (g)	5.59±15.28	3.61±12.20	4.65±12.22	8.88±20.17
Caffeine (mg)	134.89±147.98	120.28±137.02	140.66±153.40	148.66±155.57

Values are means ± SDs for continuous variables

a: moderate recreational activities for at least 10 minutes continuously in a typical week.

AF: the Animal Fluency, CERAD-DR: the Consortium to Establish a Registry for Alzheimer’s Disease Delayed Recall, CERAD-WL: the Consortium to Establish a Registry for Alzheimer’s Disease Word Learning, DSST: Digit Symbol Substitution Test.

### Logistic regression

Overall, the results were similar across the three models, while the ORs were attenuated when more covariates were included in the model. In multivariable logistic analysis (model 3), the multivariate-adjusted ORs (95% CIs) of cognitive impairment for T3 vs. T1 of serum iron concentrations were 0.70 (0.49–1.00) for DSST (*P*_for trend_ = 0.045), 0.88 (0.65–1.20) for CERAD-WL (*P*_for trend_ = 0.42), 0.65 (0.48–0.88) for CERAD-DR (*P*_for trend_<0.01) and 0.78 (0.53–1.15) for AF (*P*_for trend_ = 0.20). The inverse associations between serum iron concentrations and cognitive impairment evaluated by DSST [0.55 (0.33–0.92), *P*_for trend_ = 0.02] and CERAD-DR [0.57 (0.32–1.00), *P*_for trend_ = 0.06] were observed in men, while no association was found in women (Table 2). However, the interaction with sex was not significant in any of the analysis (all *P* values > 0.05).

**Table 2:** Odds ratio (95% confidence intervals) of cognitive impairment by tertiles of serum iron concentrations.

	Overall			Men			Women		
Cognitive test	T1	T2	T3	T1	T2	T3	T1	T2	T3
DSST<34									
Model 1	1.00	0.60 (0.42–0.86)**	0.56 (0.43–0.74)**	1.00	0.76 (0.55–1.07)	0.58 (0.37–0.92)*	1.00	0.73 (0.47–1.13)	0.63 (0.40–1.00)*
Model 2	1.00	0.61 (0.41–0.91)*	0.63 (0.44–0.90)*	1.00	0.75 (0.49–1.15)	0.52 (0.32–0.85)*	1.00	0.81 (0.53–1.24)	0.83 (0.49–1.42)
Model 3	1.00	0.65 (0.44–0.98)*	0.70 (0.49–1.00)*	1.00	0.80 (0.51–1.25)	0.55 (0.33–0.92)*	1.00	1.11 (0.63–1.95)	1.03 (0.57–1.88)
CERAD-WL<17									
Model 1	1.00	0.81 (0.64–1.04)	0.76 (0.58–0.99)*	1.00	0.97 (0.70–1.35)	0.88 (0.63–1.22)	1.00	0.68 (0.44–1.05)	0.65 (0.46–0.93)*
Model 2	1.00	0.92 (0.73–1.18)	0.86 (0.65–1.15)	1.00	1.02 (0.71–1.45)	0.86 (0.60–1.25)	1.00	0.82 (0.51–1.33)	0.78 (0.53–1.14)
Model 3	1.00	0.96 (0.73–1.26)	0.88 (0.65–1.20)	1.00	1.02 (0.69–1.51)	0.83 (0.55–1.25)	1.00	0.98 (0.59–1.61)	0.86 (0.57–1.31)
CERAD-DR<5									
Model 1	1.00	0.75 (0.53–1.07)	0.60 (0.46–0.80)**	1.00	1.07 (0.69–1.65)	0.67 (0.42–1.07)	1.00	0.97 (0.69–1.38)	0.65 (0.43–0.97)*
Model 2	1.00	0.83 (0.57–1.21)	0.65 (0.49–0.86)**	1.00	1.18 (0.74–1.89)	0.68 (0.40–1.15)	1.00	1.11 (0.77–1.59)	0.73 (0.49–1.08)
Model 3	1.00	0.87 (0.60–1.26)	0.65 (0.48–0.88)**	1.00	1.16 (0.73–1.84)	0.57 (0.32–1.00)*	1.00	1.32 (0.92–1.91)	0.79 (0.51–1.22)
AF<14									
Model 1	1.00	0.65 (0.48–0.89)**	0.70 (0.49–1.00)	1.00	1.01 (0.62–1.63)	0.92 (0.52–1.63)	1.00	0.77 (0.56–1.07)	0.71 (0.49–1.04)
Model 2	1.00	0.70 (0.52–0.96)*	0.75 (0.52–1.09)	1.00	1.05 (0.62–1.78)	0.96 (0.51–1.84)	1.00	0.91 (0.68–1.21)	0.80 (0.55–1.17)
Model 3	1.00	0.74 (0.53–1.02)	0.78 (0.53–1.15)	1.00	1.15 (0.69–1.92)	1.00 (0.52–1.93)	1.00	0.96 (0.67–1.37)	0.82 (0.54–1.26)

Model 1 was adjusted for data release cycle, age group, sex, race/ethnicity and body mass index.

Model 2 was adjusted for covariates in model 1, and also poverty-income ratio, education and marital status.

Model 3 was adjusted for covariates in model 2, and also hypertension, diabetes, moderate recreational activities, serum cotinine and daily intakes of sugar, fat, protein, caffeine, alcohol and total energy.

T1: tertile 1, T2: tertile 2, T3: tertile 3.

*: P<0.05

**: P<0.01.

AF: the Animal Fluency, CERAD-DR: the Consortium to Establish a Registry for Alzheimer’s Disease Delayed Recall, CERAD-WL: the Consortium to Establish a Registry for Alzheimer’s Disease Word Learning, DSST: Digit Symbol Substitution Test.

### Restricted cubic splines

**Fig 1:**
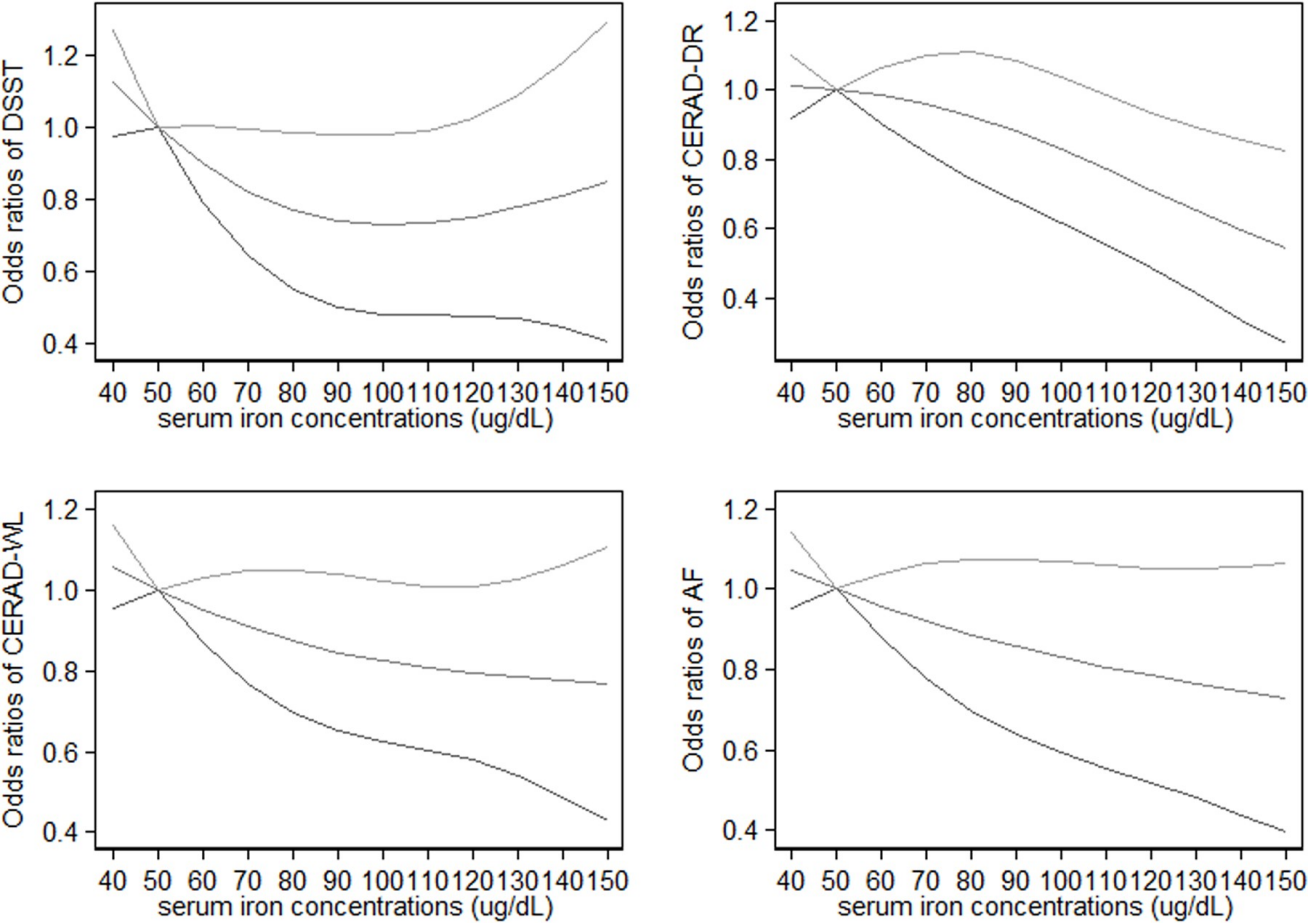
Dose-response relationship between serum iron concentrations and the odds of scoring low on the DSST, CERAD-DR, CERAD-WL and AF in older adults, respectively. The middle line and upper and lower line represent the estimated odds ratio and its 95% confidence interval, respectively. AF: the Animal Fluency, CERAD-DR: the Consortium to Establish a Registry for Alzheimer’s Disease Delayed Recall, CERAD-WL: the Consortium to Establish a Registry for Alzheimer’s Disease Word Learning, DSST: Digit Symbol Substitution Test.

We observed a steep significant association with a decrease in odds of cognitive impairment evaluated by DSST up to 90 ug/dL of serum iron concentrations [OR (95% CI): 0.74 (0.50–0.99), after which the curve almost reached a plateau. However, the departure from a linear relationship between serum iron concentrations and cognitive impairment evaluated by DSST was not significant (*P*_for non-linearity_ = 0.16), which was caused by the relatively wide range of the 95% CIs. The odds of cognitive impairment evaluated by CERAD-DR decreased linearly (*P*_for non-linearity_ = 0.35) with increasing levels of serum iron concentrations, with an apparent association for serum iron concentrations around 110 ug/dL [OR (95% CI): 0.77 (0.56–0.98)]. No association was found between serum iron concentrations and cognitive impairment evaluated by CERAD-WL and AF, respectively (Fig 1).

## Discussion

In this study, higher serum iron concentrations were inversely associated with cognitive impairment evaluated by DSST and CERAD-DR among older adults, and the associations were linear. In addition, the inverse associations were mainly observed in men but not in women.

The importance of iron in disease processes and normal function of the brain has been summarized elsewhere [4, 5]. Iron is a cofactor of ribonucleotide reductase, which is responsible for the rate limiting step of DNA synthesis, making iron essential for cell division and neural tube formation [5, 16]. Beyond cell division, neurons need timely and adequate iron supply for neurotransmitter synthesis, synapse formation, and dendritic arborization [5]. In addition, polymorphisms in iron regulating genes like HFE have significant clinical and pathophysiological impact in the nervous system [5]. In addition, previous data indicated that iron deficiency has detrimental effects on cardiovascular diseases including coronary artery disease, heart failure and pulmonary hypertension [17], and a higher cardiovascular health score were associated with a lower risk of dementia and lower rates of cognitive decline in a recent study [18]. Excess redox-active iron can also lead to oxidative damage and cell death [5]. Ferroptosis is an intracellular iron-dependent cell death pathway, and is characterized by the overwhelming, iron-dependent accumulation of lethal lipid reactive oxygen species [5, 19]. However, in the dose-response analysis, we did not observe detrimental effects of higher serum iron on cognitive impairment within the concentrations observed in this study.

Epidemiological evidence on peripheral iron and cognitive impairment incidence in older adults is limited, and the results were variable across studies [12]. Elevated transferrin saturation was not associated with the risk of developing Alzheimer’s disease in US adults followed from baseline in 1971–1974 to 1992 [20]. In another study, elevated serum iron levels may decrease cognitive speed in older individuals susceptible to cognitive impairment assessed by hemochromatosis C282Y genotype [21]. The trend suggested that higher iron intake maybe associated with a decreased risk of cognitive impairment in women and an increased risk in men [22]. Higher iron content in the caudate nucleus predicted lesser improvement in working memory after repeat testing in 78 adults from Metro Detroit area [23]. Low levels of hemoglobin but not dietary iron intake were associated with increased risk of mortality from Alzheimer’s disease [24]. An increased risk of decline was associated with higher levels of cerebrospinal fluid ferritin level among individuals of apolipoprotein Ɛ4 allele [25]. Therefore, the diversity in iron measures, cognitive outcomes and main findings precludes any conclusions relating to the relationship between peripheral iron and cognitive impairment incidence. A previous study from NHANES showed that total iron intake was inversely associated with cognitive impairment assessed by DSST, CERAD and AF in unadjusted model; however, the association was significant only in the analysis with DSST test after adjusting for other covariates [13]. In addition to iron, several other metals such as copper, zinc and manganese are also essential cofactors for many cellular enzymes, and were also found to be associated with cognitive impairment in NHANES. Dietary intakes of zinc, copper, selenium and magnesium were found inversely associated with cognitive impairment [13, 26]. In addition, blood selenium, copper and zinc were also inversely associated with cognitive impairment [27, 28]. However, blood cadmium was associated with worse cognitive function [29] while no association was found between blood lead and cognitive performance [30]. The above-mentioned findings from NHANES are generally consistent from those in meta-analyses [31–34], expect for serum copper whose concentrations were higher in patients with cognitive impairment than controls [35].

In our analysis, serum iron concentrations were associated with cognitive impairment evaluated by DSST and CERAD-DR, while no association was found with cognitive impairment evaluated by CERAD-WL and AF. The CERAD test specifically assesses episodic memory, while AF test assesses verbal fluency and semantic-based memory function and DSST is a sensitive measure of frontal lobe executive function. Therefore, the reasons for the inconsistencies with different tests maybe because the cognitive domain emphasized in each test is not consistent. A randomized controlled trial in mice suggested that iron intake had a differential effect on various brain regions [36], and dysregulations in various areas could lead to different symptoms. The study by Gao et al. also showed that serum iron concentrations were only positively correlated with brain iron in the right hippocampus and were not correlated with brain iron other regions of interest [37]. A recent review summarized that iron accumulated heterogeneously across brain regions, and Caudate nuclei, Hippocampus and Thalamus were the regions where iron was most frequently reported to correlate with memory performance, while iron deposition in the putamen was correlate to poorer general cognition [7]. These findings suggested that abnormal iron status might have different effects on various brain regions.

Strengths of this study included relatively large number of participants, cognitive impairment assessed by the four commonly used separate tests and a number of covariates. In addition, the dose-response relationship between serum iron concentrations and cognitive impairment was also explored. There are also several limitations. First, only serum iron was included in this study. The critical role of brain barrier systems in maintaining brain iron homeostasis in the central nervous system has been summarized elsewhere [38]. In brief, the brain barrier systems are comprised of the blood-brain barrier and blood-cerebrospinal fluid barrier, and transferrin-bound iron is the primary species transported into the brain by the blood-brain barrier [38]. The transferrin receptors in cerebral endothelia are about 3–7 fold higher in the striatum and hippocampus than in the cortex, which explains the uneven distribution of iron in various brain regions [38]. Meanwhile, the active transport process of iron efflux from cerebrospinal fluid to blood by the blood-cerebrospinal fluid barrier enables the body to maintain a relatively stable level of iron in the brain [38]. Gao et al. found that while both brain iron deposition and body iron levels increased in patients with cognitive impairment, serum iron in patients was only positively correlated with iron content in the right hippocampus (*P* = 0.04) [37]. In the Austrian Stroke Prevention Study, serum iron levels were not significant determinants of brain iron accumulation in normally aging subjects [39]. Furthermore, in the case of the iron-trafficking disease, iron elevation in tissues may accompany low iron markers in the blood because iron cannot efficiently be exported out of the cell [40]. These findings suggested that brain iron measures are not necessarily associated with peripheral iron, and may also explain the previous inconsistent findings between brain iron and peripheral iron and cognitive impairment. Our results are comparable with those from the NHANES study on dietary iron intake and cognitive impairment [13], which is consistent with the fact that serum iron levels are more dependent on dietary iron intake than brain iron concentrations. Second, although we adjusted for a number of covariates, residual confounding owing to measurement error or unmeasured confounding could be of concern. However, the results were generally comparable across the three statistical models in this study. Third, reverse causality should be considered because of the cross-sectional design. However, there are no clinical guidelines to date recommending or limiting iron intake for prevention of cognitive impairment. Finally, cognitive assessments cannot replace a diagnosis based on a clinical examination; however, they are useful to examine the associations of cognitive functioning with many medical conditions and risk factors in NHANES [13, 14, 41].

In summary, higher serum iron concentrations were inversely associated with cognitive impairment evaluated by DSST and CERAD-DR among older adults. However, given the consistent correlation between cognitive dysfunction and iron deposition in brain, our findings indicated that iron deposition in brain is not necessarily associated with peripheral iron concentrations, and our results should be interpreted cautiously. The relationships between peripheral iron concentrations and cognitive impairment deserve to be confirmed by longitudinal studies.

## Supporting information

S1 Table STROBE statement checklist of items that should be included in reports of observational studies. (DOC)
