# Supplementary material for: Association between serum iron concentrations and cognitive impairment in older adults aged 60 years and older: A dose-response analysis of National Health and Nutrition Examination Survey
Source: PLoS One. 2021 Aug 2;16(8):e0255595. doi: 10.1371/journal.pone.0255595 (PMC8328322; doi:10.1371/journal.pone.0255595)
Supplement: S1 Table — (DOC) [file pone.0255595.s001.doc]

S1 Table

STROBE statement checklist of items that should be included in reports of observational studies

|  | **Page** | **Item No** | **Recommendation** |
| --- | --- | --- | --- |
| **Title and abstract** |  |  |  |
|  | 1  2 | 1 | (*a*) Indicate the study's design with a commonly used term in the title or the abstract |
|  |  |  | (*b*) Provide in the abstract an informative and balanced summary of what was done and what was found |
| **Introduction** |  |  |  |
| Background/rationale | 3 | 2 | Explain the scientific background and rationale for the investigation being reported |
| Objectives | 3 | 3 | State specific objectives, including any prespecified hypotheses |
| **Methods** |  |  |  |
| Study design | 4 | 4 | Present key elements of study design early in the paper |
| Setting | 4 | 5 | Describe the setting, locations, and relevant dates, including periods of recruitment, exposure, follow-up, and data collection |
| Participants | 4 | 6 | (*a*) *Cohort study*?Give the eligibility criteria, and the sources and methods of selection of participants. Describe methods of follow-up*Case-control study*?Give the eligibility criteria, and the sources and methods of case ascertainment and control selection. Give the rationale for the choice of cases and controls*Cross sectional study*?Give the eligibility criteria, and the sources and methods of selection of participants |
|  |  |  | (*b*) *Cohort study*?For matched studies, give matching criteria and number of exposed and unexposed*Case-control study*?For matched studies, give matching criteria and the number of controls per case |
| Variables | 4-5 | 7 | Clearly define all outcomes, exposures, predictors, potential confounders, and effect modifiers. Give diagnostic criteria, if applicable |
| Data sources/ measurement | 4 | 8* | For each variable of interest, give sources of data and details of methods of assessment (measurement). Describe comparability of assessment methods if there is more than one group |
| Bias | 4-6 | 9 | Describe any efforts to address potential sources of bias |
| Study size | 4-7 | 10 | Explain how the study size was arrived at |
| Quantitative variables | 6 | 11 | Explain how quantitative variables were handled in the analyses. If applicable, describe which groupings were chosen and why |
| Statistical methods | 6 | 12 | (*a*) Describe all statistical methods, including those used to control for confounding |
|  | 6 |  | (*b*) Describe any methods used to examine subgroups and interactions |
|  | 6 |  | (*c*) Explain how missing data were addressed |
|  | 6 |  | (*d*) *Cohort study*?If applicable, explain how loss to follow-up was addressed*Case-control study*?If applicable, explain how matching of cases and controls was addressed*Cross sectional study*?If applicable, describe analytical methods taking account of sampling strategy |
|  | 6 |  | (*e*) Describe any sensitivity analyses |
| **Results** |  |  |  |
| Participants | 7 | 13* | (*a*) Report numbers of individuals at each stage of study?eg numbers potentially eligible, examined for eligibility, confirmed eligible, included in the study, completing follow-up, and analysed |
|  | 7 |  | (*b*) Give reasons for non-participation at each stage |
|  | 7 |  | (*c*) Consider use of a flow diagram |
| Descriptive data | 7-9 | 14* | (*a*)Give characteristics of study participants (eg demographic, clinical, social) and information on exposures and potential confounders |
|  | 7-9 |  | (*b*) Indicate number of participants with missing data for each variable of interest |
|  |  |  | (*c*) *Cohort study*?Summarise follow-up time (eg average and total amount) |
| Outcome data |  | 15* | *Cohort study*?Report numbers of outcome events or summary measures over time |
|  |  |  | *Case-control study?*Report numbers in each exposure category, or summary measures of exposure |
|  | 7 |  | *Cross sectional study?*Report numbers of outcome events or summary measures |
| Main results | 10-12 | 16 | (*a*) Report the numbers of individuals at each stage of the study?eg numbers potentially eligible, examined for eligibility, confirmed eligible, included in the study, completing follow-up, and analysed |
|  | 10-12 |  | (*b*) Give reasons for non-participation at each stage |
|  | 10-12 |  | (*c*) Consider use of a flow diagram |
| Other analyses | 13 | 17 | Report other analyses done?eg analyses of subgroups and interactions, and sensitivity analyses |
| **Discussion** |  |  |  |
| Key results | 13 | 18 | Summarise key results with reference to study objectives |
| Limitations | 16-18 | 19 | Discuss limitations of the study, taking into account sources of potential bias or imprecision. Discuss both direction and magnitude of any potential bias |
| Interpretation | 14-16 | 20 | Give a cautious overall interpretation of results considering objectives, limitations, multiplicity of analyses, results from similar studies, and other relevant evidence |
| Generalisability | 18 | 21 | Discuss the generalisability (external validity) of the study results |
| **Other information** |  |  |  |
| Funding |  | 22 | Give the source of funding and the role of the funders for the present study and, if applicable, for the original study on which the present article is based |
